# A multi-dimensional feature aggregation network for electric vehicle charging demand prediction

**DOI:** 10.1038/s41598-026-38855-3

**Published:** 2026-03-11

**Authors:** Yi Yu, Lihua He, Ziyue Yu, Yanqiang Tu, Xiaozhu Jing, Wuman Luo

**Affiliations:** 1 Faculty of Applied Sciences, Macao Polytechnic University, Macao SAR, China; 2 School of Mechanical and Electrical Engineering, Zhongshan Institute, University of Electronic Science and Technology of China, Zhongshan, 528402 China

**Keywords:** Engineering, Mathematics and computing

## Abstract

Accurate prediction of urban electric vehicle (EV) charging demand is critical for infrastructure planning and dynamic pricing strategies. Although various methods have been developed, most existing studies focus primarily on spatiotemporal dependencies, paying limited attention to interactions among multivariate features. Furthermore, conventional serial spatiotemporal architectures typically extract features dimension-by-dimension, which may impede cross-dimensional information flow and lead to imbalanced representations. To address these challenges, we propose the Multi-Dimensional Feature Aggregation Network (MDFANet). MDFANet is designed to enhance multivariate representations while embedding spatiotemporal attention to strengthen relational modeling. Specifically, we introduce a Multi-Dimensional Feature Aggregation Module (MDFAM) that conducts fine-grained aggregation along both temporal and variable dimensions. By fusing these aggregated features with raw inputs, the model preserves distributional and semantic heterogeneity. Extensive experiments on real-world datasets demonstrate that MDFANet outperforms competitive baselines in prediction accuracy while reducing computational costs by approximately 50%. For reproducibility, the source code is available at https://github.com/kion-86/MDFANet.

## Introduction

With the rapid global adoption of electric vehicles (EVs), the EV market has experienced exponential growth. According to the International Energy Agency (IEA)^1^, global electric car sales exceeded 17 million in 2024, accounting for over 20% of the market share. As EV charging constitutes a substantial and increasing proportion of urban electricity consumption^2–5^, accurate demand prediction has become essential for both planning and operational decision-making^6^. Reliable forecasting facilitates critical applications such as charging station planning, grid load balancing, and smart infrastructure management^7–12^, including scheduling-aware coordination in practical systems. Moreover, precise demand prediction allows EV users to optimize travel schedules and charging plans, thereby minimizing unnecessary wait times.

**Fig. 1:**
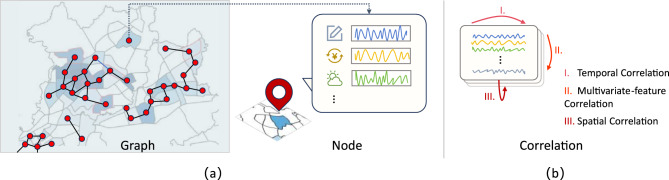
Illustration of data structure and multi-dimensional correlations in urban EV charging demand prediction. (**a**) The city is partitioned into regions to form a graph structure, where each region (node) contains multivariate time-series features. (**b**) Three types of key correlations: temporal correlation, multivariate-feature correlation, and spatial correlation.

However, predicting EV charging demand remains challenging due to the complex dependencies within charging data. As illustrated in Fig. 1, the data exhibit high interdependence across spatial, temporal, and multivariate dimensions. Typically, these correlations fall into three categories: (1) Spatial correlation, where charging demands in adjacent zones or functionally linked stations influence one another; (2) Temporal correlation, characterized by positive correlations in adjacent time intervals and distinct daily or weekly seasonality; and (3) Multivariate-feature correlation, where exogenous variables—such as electricity prices, weather conditions, calendar effects, and traffic mobility—are associated with charging demand and may modulate its dynamics.

Deep learning-based time-series models have been widely adopted for EV charging prediction due to their ability to capture temporal dependencies^13,14^. While effective in handling temporal sequences, standard time-series models often neglect spatial relationships between urban regions, which limits their predictive potential^15^. Consequently, graph-structured models, particularly Graph Neural Networks (GNNs), have been introduced to model spatial topology. Following the success of the Transformer architecture^16^, researchers have further incorporated attention mechanisms into spatiotemporal frameworks to enhance accuracy^17,18^. More recently, physics-informed methods have been integrated into spatiotemporal models. While these approaches often surpass purely data-driven models in accuracy and interpretability, they typically rely on direct inputs of raw spatiotemporal data. This dependency may overlook underlying multi-dimensional feature interactions, potentially constraining model applicability in complex scenarios^19,20^. Furthermore, the serial structure of traditional spatiotemporal modules may attenuate temporal information during spatial feature extraction, thereby reducing the efficiency of subsequent time-series modeling^21^. Thus, while physics-informed and attention-based models achieve accuracy gains, they often do so at the cost of increased model complexity, requiring substantial computational resources and extended training times for large-scale datasets^22,23^.

To address the aforementioned trade-off between accuracy and efficiency, we propose the Multi-Dimensional Feature Aggregation Network (MDFANet). This approach is designed to capture fine-grained dependencies among multiple variables in EV charging data. We introduce a time-series feature enhancement module prior to the spatiotemporal module to improve the quality of feature representations. Unlike traditional approaches that model features in a single dimension, our proposed Multi-Dimensional Feature Aggregation Module (MDFAM) comprises two key components: the Feature Core Generator (FCG) and Feature Fusion (FF). The FCG performs aggregation along both variable and temporal axes, synthesizing a “feature core” that encapsulates multivariate dependencies. The FF then injects this feature core back into the input space, preserving distributional heterogeneity while reinforcing key information.

The main contributions of this paper are as follows:

1. We propose MDFANet, a comprehensive framework that jointly models spatial, temporal, and multivariate-feature correlations to accurately predict urban EV charging demand.
2. We introduce a novel spatiotemporal data processing framework that enhances time-series features via aggregation before spatiotemporal modeling, thereby improving the overall robustness of feature representation.
3. We design the Multi-Dimensional Feature Aggregation Module (MDFAM), which performs fine-grained aggregation across variable and time dimensions. This module effectively fuses aggregated features with original data to maintain semantic heterogeneity while strengthening signal representation.
4. We conduct extensive experiments on real-world datasets. The results demonstrate that MDFANet achieves an average improvement of 4.48% across four key performance metrics compared to the state-of-the-art physics-informed model (PIAST), while reducing training time by approximately 51%.

The remainder of this paper is organized as follows: “Related work” reviews related work. “Methodology” details the methodology of the proposed MDFANet. “Experiment” presents the experimental results and discussion. Finally, “Conclusion” concludes the paper.

## Related work

Some early approaches to EV charging demand prediction research were based on data-driven prediction methods. Data-driven prediction methods are a class of techniques that utilize a large amount of historical data and algorithmic models for prediction and inference^24,25^. Compared to traditional methods that rely on explicit physical models or human rules, data-driven methods predict future behaviors or changes by learning from historical data and identifying underlying patterns, trends, and relationships. In the early stages of EV charging demand prediction^26^ , research relied heavily on time-series data for modeling. Predictions of charging demand are usually modeled with statistical methods^27,28^. For example, quantifying the charging station load characteristics through the maximum mutual information coefficient (MIC) and combining it with the Least Absolute Shrinkage and Selection Operator regression model (LASSO) for feature selection has achieved some success in demand modeling, but it fails to account for complex external factor interference^29^. Another way is to decouple EV charging demand from traditional electric loads using ARIMA to improve accuracy, but it still fails to account for the influence of external perturbations^30^.

With the improvement of nonlinear modeling capability, machine learning methods have gradually been introduced into the EV charging demand prediction task. Support vector regression (SVR)^31^ models combined with feature engineering methods improves the ability to capture complex nonlinear relationships by extracting load characteristics, weather variables, time attributes, and holiday factors^32^. Random forest (RF) have been used to classify the usage status of charging stations, achieving high prediction accuracy^33,34^. However, traditional machine learning methods face performance bottlenecks when dealing with large-scale data and highly nonlinear relationships, prompting research to further turn to deep learning methods. Relying on its ability to capture complex spatio-temporal and nonlinear relationships, deep learning techniques demonstrate stronger generalization ability and prediction accuracy in EV charging demand prediction. Short-term charging demand prediction using long short-term memory neural network (LSTM) outperforms traditional statistical and shallow neural network models in terms of accuracy^35,36^. An LSTM-based two-layer architecture combined with a grid-optimization algorithm to solve the charging-station layout problem can effectively reduce power loss^37^. However, the model architecture integrating LSTM and feature selection methods faces the problems of sensitivity to model parameters and data dependency while improving accuracy^38^. In further development, the combination of convolutional neural networks (CNNs)^39,40^ and LSTMs is used to extract spatial features and temporal dependencies to enhance the performance of time series prediction. The use of gated recurrent unit (GRU) structures and the optimization of hyperparameters in combination with genetic algorithms can achieve optimal performance among multiple deep models^41,42^. In addition, the introduction of spatio-temporal graph neural networks (e.g., STGCN) combining graph convolution and temporal convolution can effectively model the spatio-temporal dependencies in traffic timeseries data. For example, ST-RLNet ^43^ learns spatio-temporal representations to capture dynamic correlations, while graph-based frameworks ^44^ have effectively modeled spatial constraints in regional traffic. However, these methods may suffer degraded predictive performance when the data quality is insufficient. The spatio-temporal graph regression network (ASTGRN) constructed using an adaptive graph learning layer and gating mechanism further enhances the ability to model spatial relationships between nodes and temporal dynamics, but at the same time imposes a high computational burden^45^.

In contrast to purely data-driven approaches, recent research has begun to fuse physical knowledge with data models through the introduction of physics-informed neural networks (PINNs)^46,47^, which utilize the laws of physics, such as differential equations, to guide the training process of the neural networks, allowing the models to maintain higher accuracy under scenarios with incomplete data or sparse samples and Physics consistency. In charging demand modeling, the relationship between price and demand is used as a physical constraint to generate pre-trained data that conforms to the physical laws to enhance the model generalization ability. The model structure constructed by combining Graph Attention Network (GAT)^48^ and Temporal Attention Mechanism (TPA-LSTM)^49^can successively extract correlations in both spatial and temporal dimensions to realize high-accuracy prediction of charging demand in complex urban environments^50^. Although the predictive effect of this class of methods is significant, their performance relies on the clarity of the physical laws and modeling accuracy in the target task. The model architecture itself also places high demands on computational resources, with computational complexity rapidly increasing and pre-training time significantly lengthening as the node size and time window expand. The current optimal prediction framework integrates physical information and spatio-temporal graph structure, introduces feature information based on physical relationships in the model loss function, and adopts a staged training strategy: initially, emphasize the physical constraints to improve the model interpretability, and gradually shift to data-driven optimization of the target task’s performance in later stages. This hybrid strategy enhances the stability and generalization ability of the model. However, the method is more dependent on physical information and price data, which are not always available or reliable in real-world scenarios. Also, the design of the dual-attention mechanism and the physical prior fusion mechanism brings high computational and implementation complexity.

In summary, research on EV charging demand prediction has improved the accuracy of the prediction task by introducing methods such as spatial information, spatio-temporal attention relations and physical information. However, it is still a challenge to mine the information of the data itself, improve the efficiency of data feature characterization, reduce the dependence of the data on temporal attention relations, as well as solve the problems of data scarcity and robustness. Therefore, this paper proposes a novel approach to improve the efficiency of data feature characterization and reduce the data’s temporal attention dependency by aggregating the cores of different dimensional features of the data, so as to reduce the computational complexity of the model without decreasing the model’s prediction accuracy.

## Methodology

In this section, we first formulate the problem of EV-charging demand prediction. Then we present the proposed Model in detail.

**Fig. 2:**
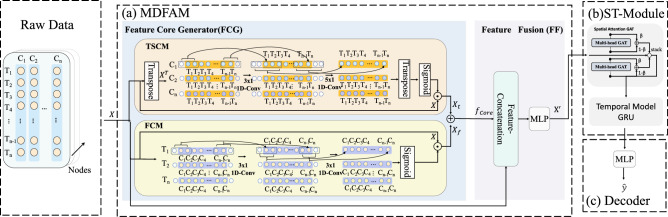
Overview of the MDFANet. The MDFANet consists of 3 components: (**a**) MDFAM, (**b**) ST-Module, (**c**) Decoder.

### Problem definition

Let $G = (V, E, A)$ denote the urban electric vehicle charging network, where *V* is a set of *N* charging zones ($|V| = N$) and *E* is the set of edges representing the connectivity between these zones. The topological structure is represented by a static adjacency matrix $A \in \mathbb {R}^{N \times N}$. Let $X_t \in \mathbb {R}^{N \times F}$ denote the node feature matrix at timestep *t*, where *F* represents the feature dimension. Given the graph *G* and a sequence of historical observations with a window size of *T*, denoted as $\textbf{X}_{t-T:t-1} = \{ X_{t-T}, X_{t-T+1}, \dots , X_{t-1} \} \in \mathbb {R}^{T \times N \times F}$, we aim to learn a mapping function $f(\cdot )$ that predicts the electric vehicle charging demand at the *L*-th future timestep (i.e., timestep $t+L-1$):

1 $$\begin{aligned} & \ \hat{Y}_{t+L-1} = f(\textbf{X}_{t-T:t-1}; \theta , G) & \end{aligned}$$

where $\theta$ denotes all the learnable parameters, $Y_{t+L-1} \in \mathbb {R}^{N}$ denotes the ground-truth charging-demand vector and $\hat{Y}_{t+L-1} \in \mathbb {R}^{N}$ denotes the corresponding prediction.

### MDFANet

As illustrated in Fig. 2, the proposed approach consists of three modules, namely (a) a multi-dimensional feature aggregation module, which enhances the representation of data features through multi-dimensional feature aggregation; (b) a spatio-temporal module, which extracts spatio-temporal relational features via a spatio-temporal module; and (c) a decoder module, which transforms the final features into the predicted targets. Details of each module are described as follows.

#### Multi-dimensional feature aggregation module (MDFAM)

This study proposes a method to aggregate core features of different dimensions and perform feature enhancement on raw data. This approach enhances the feature representation of the data, improves the robustness of the data representation, and strengthens the efficiency of the subsequent spatio-temporal module. The method mainly includes two core parts: Feature Core Generator and Feature Fusion.

Feature Core Generator(FCG): Feature Core Generator consists of three parts: a timeseries convolution module, a feature convolution module, and a feature core aggregation module. It uses the first two modules to separately extract temporal-core and feature-core representations within the embedding module. These two cores are fused by element-wise addition as a residual-style operation to keep the embedding stage lightweight and stable, introducing minimal extra parameters and computation while maintaining reproducibility. The downstream GAT and GRU modules subsequently provide dedicated spatial and temporal modeling capacity, and the fused embedding is then fed into the aggregation module to generate the feature core.

Time Series Convolution Module (TSCM): This module performs one-dimensional convolution along the temporal axis to capture local temporal patterns. Given the input of node $i$, $x_i \in \mathbb {R}^{T \times F}$ , where $T$ denotes the length of the time window and $F$ the number of features per time step, the two-layer temporal convolution is defined as:

2 $$\begin{aligned} T_{i,1}&= W_{t1} *_{t}\, x_i^\top + b_{\textrm{tem},1} \end{aligned}$$

3 $$\begin{aligned} T_{i,2}&= W_{t2} *_{t}\, T_{i,1} + b_{\textrm{tem},2} \end{aligned}$$

where $x_i^\top$ is the transpose of $x_i$ (i.e., $x_i^\top \in \mathbb {R}^{F \times T}$); $T_{i,1}, T_{i,2} \in \mathbb {R}^{F \times T}$ represent the intermediate feature sequences along the entire time axis (with the same time window length $T$ as input) produced by the first and second temporal convolution layers, respectively; $W_{t1} \in \mathbb {R}^{F \times F \times 3}$ and $W_{t2} \in \mathbb {R}^{F \times F \times 5}$ are the weights of the two convolution kernels with kernel lengths 3 and 5, respectively; $b_{\textrm{tem},1}, b_{\textrm{tem},2} \in \mathbb {R}^{F}$ are bias parameters (one per output feature channel); the operator $*_{t}$ denotes one-dimensional convolution along the temporal dimension (the length-$T$ dimension). To preserve the time window length $T$ after convolution, the first layer uses padding=1 and the second layer uses padding=2. The output is the two-layer temporal convolutional result.

The output $T_{i,2}$ is first transposed to match the dimension of the original input $x_i$, then passed through the sigmoid activation function, and finally combined with $x_i$ via element-wise multiplication:

4 $$\begin{aligned} & \hat{x}_{i,t} = x_i \odot \sigma \left( T_{i,2}^\top \right) & \end{aligned}$$

where $T_{i,2}^\top$ is the transpose of $T_{i,2}$; $\sigma$ denotes the sigmoid activation function; $\odot$ represents element-wise multiplication; and $\hat{x}_{i,t} \in \mathbb {R}^{T \times F}$ is the final output of the TSCM, with the same dimension as the input $x_i$.

Feature Convolution Module (FCM): The feature convolution module facilitates information integration across features by performing 1D convolution along the feature dimension through two layers. Given the input data $x_i \in \mathbb {R}^{T \times F}$, the operations of the module are expressed as:

5 $$\begin{aligned} F_{i,1}&= W_{f1} *_{f}\, x_i + b_{\textrm{feat},1} \end{aligned}$$

6 $$\begin{aligned} F_{i,2}&= W_{f2} *_{f}\, F_{i,1} + b_{\textrm{feat},2} \end{aligned}$$

where $F_{i,1}, F_{i,2} \in \mathbb {R}^{T \times F}$ are the intermediate feature sequences produced by the first and second convolution layers, respectively; $W_{f1}, W_{f2} \in \mathbb {R}^{T \times T \times 3}$ are the convolution kernels with kernel size 3; $b_{\textrm{feat},1}, b_{\textrm{feat},2} \in \mathbb {R}^T$ are the bias parameters; and $*_{f}$ denotes 1D convolution along the feature dimension. Both layers use padding=1 to preserve the number of features F.

The output of the second convolution layer is passed through a sigmoid activation function, and then element-wise multiplication is performed with the original input to generate the final output:

7 $$\begin{aligned} & \hat{x}_{i,f} = x_i \odot \sigma (F_{i,2}) & \end{aligned}$$

where $\sigma$ is the sigmoid activation function; $\odot$ denotes element-wise multiplication; and $\hat{x}_{i,f} \in \mathbb {R}^{T \times F}$ is the final output of the FCM, with the same dimension as the input $x_i$.

Feature core extraction: The output from the time series convolution module (TSCM) and the output from the feature convolution module (FCM) are combined via element-wise addition to form the feature core $f_{\text {core}} \in \mathbb {R}^{T \times F}$:

8 $$\begin{aligned} & f_{\text {core}} = \hat{x}_{i,f} + \hat{x}_{i,t} & \end{aligned}$$

Feature fusion (FF): To fully leverage the complementary information between the original input and the extracted feature core, the Feature Fusion module implements a structured transformation process to generate discriminative and informative features that capture key temporal patterns and cross-feature correlations. Specifically, we first concatenate the original input data $x_i \in \mathbb {R}^{T \times F}$ and the feature core $f_{\text {core}} \in \mathbb {R}^{T \times F}$ along the feature dimension, resulting in a concatenated feature map $f_{\text {cat}} \in \mathbb {R}^{T \times 2F}$ that aggregates both raw and refined feature information. Next, the concatenated features are fed into a two-layer fully connected network with a GELU activation function to realize dimension reduction and non-linear feature enhancement. Finally, the transformed features are mapped to the target output space to complete the feature fusion process. The mathematical formulation of this process is as follows:

9 $$\begin{aligned} f_{\textrm{cat}}&= \text {concat}(x_i, f_{\text {core}}) \end{aligned}$$

10 $$\begin{aligned} h&= \text {GELU}(f_{\textrm{cat}} \times W_{F1} + b_{F1}) \end{aligned}$$

11 $$\begin{aligned} x_i'&= h \times W_{F2} + b_{F2} \end{aligned}$$

where $W_{F1} \in \mathbb {R}^{2F \times F}$ and $b_{F1} \in \mathbb {R}^F$ denote the weight matrix and bias vector of the first fully connected layer; $W_{F2} \in \mathbb {R}^{F \times F}$ and $b_{F2} \in \mathbb {R}^{F}$ are the weight matrix and bias vector of the second fully connected layer (mapping the enhanced features to the final output dimension); and GELU activation is adopted to introduce non-linearity and mitigate gradient vanishing issues.

By integrating the feature core aggregation and the above feature fusion mechanism, the MDFAM module supports modeling temporal dependencies in sequential data and cross-feature associations. This enables the model to generate robust and comprehensive feature representations, laying a solid foundation for accurate time-series analysis and prediction tasks.

#### Spatio-temporal module

In order to better extract the relationship between different regions, the GAT algorithm based on the multi-head attention mechanism is used to aggregate the features of neighboring nodes with high relevance. The spatial Feature Extraction Module is composed of multiple GAT layers.The input of each GAT layer is the node features (i.e., $X' = \{x_1', x_2', \dots , x_N'\}$, where $x_i' \in \mathbb {R}^{F}$, $N$ is the number of nodes, and $F$ is the feature number of each node), and the output is the neighbor-infused Features (i.e., $X'' = \{x_1'', x_2'', \dots , x_N''\}$, where $x_i'' \in \mathbb {R}^{F' }$) .

The first step is to calculate the attention coefficient for each neighbor $j \in N_i$ of node i, which is calculated according to Eq. (12).

12 $$\begin{aligned} & \alpha _{ij} = softmax(\frac{\exp \left( \text {LeakyReLU} \left( \textbf{a}^T \left[ W\mathbf {x'}_i \parallel W\mathbf {x'}_j \right] \right) \right) }{\sum _{k \in N_i} \exp \left( \text {LeakyReLU} \left( \textbf{a}^T \left[ W\mathbf {x'}_i \parallel W\mathbf {x'}_k \right] \right) \right) }) & \end{aligned}$$

where $\alpha _{ij}$ denotes the attention coefficient between nodes *i* and *j*. The symbol $\Vert$ represents the concatenation operation. $W \in \mathbb {R}^{F' \times F}$ refers to a shared linear transformation applied to each node, which adjusts the number of input features from *F* to $F'$. Furthermore, $a: \mathbb {R}^{F'} \times \mathbb {R}^{F'} \rightarrow \mathbb {R}$ represents the attention mechanism, which utilizes the Leaky ReLU activation function to process the concatenated high-dimensional features. The attention coefficient indicates the feature importance between nodes. The softmax function is used to normalize it.

The second step is to perform *K* independent attention mechanisms for node *i*, and average them to generate the output $x''_i$ of the GAT layer according to Formula (13):

13 $$\begin{aligned} & x_i'' = \sigma \left( \frac{1}{K} \sum _{k=1}^{K} \sum _{j \in \mathcal {N}_i} \alpha _{ij}^{k} W^k x'_j \right) & \end{aligned}$$

where $\alpha _{ij}^k$ are normalized attention coefficients computed by the $k$-th attention mechanism, and $W^k$ is the corresponding input linear transformation’s weight matrix, $\sigma$ is the activation function (e.g., Sigmoid, ReLU, etc.).It is important to note that, in this context, the final output $x_i''$ will contain $K F'$ features per node.

The spatial information of nodes can be captured and propagated to their neighboring nodes by stacking multiple GAT layers. For instance, when M GAT layers are employed, each node can acquire information from its M-hop neighbors. To address the over-smoothing issue caused by the stacking of GAT layers, a momentum residual connection is introduced with each GAT layer. Specifically, for node i, the final output of Spatial Feature Extraction Module, denoted as $x_i^{GAT}$ , is computed according to Formula (14).

14 $$\begin{aligned} & x_i^{GAT} = \parallel _{k=1}^{K} \left[ (1 - \beta ) x_i''^{(k)} + \beta x_i''^{(k-1)} \right] & \end{aligned}$$

where $x_i^{GAT}$ has $KF'$ features , $K$ is the number of attention heads,$F'$ is the feature dimention after linear transformation, and $\beta$ is the residual coefficient. The feature dimensions before and after GAT are set to be the same to satisfy the residual connection constraint.

Then the output of Spatial Feature Extraction Module will be sent to the temporal Module (GRU layer) to obtain the hidden representations $h_{i,t}$ of node i:

15 $$\begin{aligned} & h_{i,t} = \text {GRU}(h_{i,t-1},x_i^{GAT}) & \end{aligned}$$

where $h_{i,t-1}$ is the hidden representation of time step $t-1$ of node i.

#### Decoder

Finally, the prediction result $\hat{y_i}$ is calculated as follows:

16 $$\begin{aligned} & \hat{y_i} = \sigma (W_1 \cdot \text {LeakyReLU}(W_0 \cdot h_{i,t} + b_0) + b_1) & \end{aligned}$$

where $W_0$, $W_1$, $b_0$ and $b_1$ are the weight matrices and biases, respectively.

## Experiment

In this section, the proposed method is evaluated on an electric vehicle (EV) charging dataset using four metrics, and its performance is compared with the PIAST model and other representative models. Ablation experiments are conducted to assess the contribution of each component of the model. Subsequently, the training set is perturbed with missing and erroneous data, and the model is trained on these processed datasets to evaluate its robustness.

### Experiment settings

#### Dataset

The ST-EVCDP is a public dataset^1^. It contains EV charging data from 247 zones in Shenzhen, from 19 June to 18 July 2022. The dataset includes charging demand and real-time pricing collected every five minutes for each zone. The evaluation data consist of 8640 timestamps, divided into training and test sets in chronological order with an 8:2 ratio. The task is to predict charging demand at four future time points (15, 30, 45, and 60 min ahead) based on one hour of historical data.

#### Evaluation metrics

To ensure a comprehensive evaluation of the proposed model, four widely used performance metrics are employed: Root Mean Squared Error (RMSE), Mean Absolute Percentage Error (MAPE), Relative Absolute Error (RAE), and Mean Absolute Error (MAE). These metrics assess the model’s accuracy and robustness in predicting charging demand across different time intervals. The detailed definitions and calculation formulas for each of these metrics are as follows:

17 $$\begin{aligned} \text {RMSE} = \sqrt{\frac{1}{n} \sum _{i=1}^{n} (y_i - \hat{y}_i)^2}, \text {MAPE} = \frac{1}{n} \sum _{i=1}^{n} \left| \frac{y_i - \hat{y}_i}{y_i} \right| \times 100\%, \text {RAE} = \frac{\sum _{i=1}^{n} |y_i - \hat{y}_i|}{\sum _{i=1}^{n} |y_i - \bar{y}|}, \text {MAE} = \frac{1}{n} \sum _{i=1}^{n} |y_i - \hat{y}_i|. \end{aligned}$$

These metrics are employed to evaluate the model’s predictive accuracy, robustness to variations in input data, and overall performance across different time intervals. By combining these metrics, we provide a balanced analysis of the proposed model’s predictive performance under varying conditions.

#### Compared methods

In the evaluation of the proposed model using the four aforementioned metrics, we compare its performance with the well-performing PIAST model, along with the statistical models provided in its paper, including Vector Auto-Regression (VAR), as well as spatiotemporal neural network models such as LSTM, GCN-LSTM, and PAG. We additionally include DCRNN and ASTGNN as representative spatiotemporal graph baselines, where DCRNN models diffusion-based spatial propagation with recurrent units and ASTGNN employs attention-based spatiotemporal graph learning. Furthermore, we compare the performance and computational complexity of MDFANet with PAG and PIAST by evaluating their prediction accuracy and runtime.

#### Implementation details

We implement the proposed MDFANet on PyTorch 2.5.1 and CUDA Version 11.8. The training work is done on 13th Gen Intel(R) Core(TM) i5-13600KF CPU @ 3.50 GHz(14 cores), and an NVIDIA GeForce RTX 3070 with 8G RAM. We use the Adam optimizer with learning rate set to 1e−5. Batch size is set to 512. Unless otherwise specified, we fix the random seed to 2023 for reproducibility. We train the model for a fixed maximum of $n\_epoch{=}1000$ epochs and do not use early stopping. During training, we save model checkpoints and report test performance using the checkpoint selected by the same deterministic rule for all compared methods (i.e., the checkpoint with the lowest training loss under this fixed epoch budget), without accessing the test set for model selection. We use the mean squared error (MSE) loss for optimization.To ensure robustness, we repeated the runs under the same settings and obtained consistent results, demonstrating stable behavior under the reported experimental protocol.

### Evaluation results and discussions

The performance of the evaluated methods is assessed from four perspectives: (1) forecasting accuracy, which reflects the model’s capability to predict charging demand at future time points; (2) ablation analysis, which highlights the contribution of each component within the proposed model; (3) model efficiency, which measures the ability to achieve high performance with limited computational resources and training time; and (4) performance under data scarcity, which examines how well the model maintains accuracy when the amount of training data is reduced.

**Table 1:** Performance comparison of different models.

Metric (10$^{-2}$)	RMSE					MAPE				
Model	15 min	30 min	45 min	60 min	Avg	15 min	30 min	45 min	60 min	Avg
VAR	7.45	7.91	8.32	8.58	8.07	37.00	39.82	41.97	44.93	40.93
LSTM	8.36	8.64	8.68	8.85	8.63	44.23	45.35	46.27	47.03	45.72
GCN-LSTM	8.56	8.54	8.72	8.62	8.61	47.18	48.39	47.91	47.58	47.77
DCRNN	5.76	6.35	6.87	7.21	6.55	32.53	35.77	40.40	41.30	37.50
ASTGNN	3.30	4.59	5.53	6.35	4.94	11.34	15.71	20.61	24.02	17.92
PAG	2.90	4.37	6.25	6.25	4.94	8.08	14.11	17.54	23.83	15.89
PIAST	2.89	4.34	5.28	6.15	4.67	7.97	14.12	17.63	22.84	15.64
Ours	2.70	4.04	5.00	5.88	4.41	7.89	12.55	17.22	22.90	15.14

Metric (10$^{-2}$)	RAE					MAE				
Model	15 min	30 min	45 min	60 min	Avg	15 min	30 min	45 min	60 min	Avg
VAR	40.33	42.19	43.86	45.61	43.00	5.63	5.88	6.11	6.36	6.00
LSTM	42.81	43.89	44.37	45.21	44.07	5.97	6.12	6.19	6.30	6.15
LSTMGCN	43.18	43.17	44.04	44.37	43.69	6.02	6.02	6.14	6.04	6.06
DCRNN	30.59	32.91	35.00	36.47	33.74	4.27	4.59	4.88	5.08	4.70
ASTGNN	13.90	18.91	23.92	28.23	21.24	1.94	2.64	3.34	3.93	2.96
PAG	9.66	16.24	20.98	25.24	18.03	1.35	2.26	2.92	3.51	2.51
PIAST	9.78	16.17	20.71	24.92	17.90	1.36	2.24	2.87	3.46	2.48
Ours	9.32	15.38	19.75	23.89	17.09	1.29	2.13	2.74	3.31	2.37

#### Forecasting accuracy

Table 1 summarizes the quantitative performance of MDFANet and baseline models across four metrics. In general, deep learning-based methods outperform the traditional statistical approach (VAR) in capturing complex spatiotemporal dependencies. Specifically, while VAR slightly surpasses LSTM in this specific dataset, introducing graph-based spatial modeling (e.g., GCN-LSTM) generally enhances performance. In addition, DCRNN and ASTGNN achieve competitive performance among data-driven spatiotemporal baselines, but they are still outperformed by the physics-informed models and MDFANet in our evaluation. Among the advanced baselines, physics-informed models (PAG and PIAST) demonstrate superiority over purely data-driven spatiotemporal models. Notably, PIAST, which embeds physical constraints into the loss function, yields better results than PAG’s pre-training embedding strategy.

#### Ablation analysis

To verify the contribution of the Multi-Dimensional Feature Aggregation Module (MDFAM) and the Spatial-Temporal Attention Module, we conducted ablation studies by removing the embedding component and the spatiotemporal module, respectively.

Without EMB: We removed the embedding module and directly passed the raw input through a shape alignment process to the spatiotemporal module. Specifically, we stacked different feature variables and applied convolution-based dimensionality reduction to align the input dimensions required by the subsequent spatiotemporal module.

Without ST: We removed the entire spatiotemporal module (GAT + GRU). The output from the embedding module is passed through a lightweight linear layer to directly map to the prediction space, generating $\hat{Y}$ with the same shape as the original decoder, thus isolating the contribution of the spatiotemporal module.

The results in Table 2 indicate that removing either component leads to performance degradation. Specifically, the full MDFANet reduces RMSE by approximately 3.17% and MAPE by 7.20% on average compared to its ablation variants. The performance drop in the *without EMB* variant suggests that the multidimensional feature aggregation is critical for enhancing data representation. Similarly, the decline in the *without ST* variant confirms the necessity of the attention mechanism in capturing long-range temporal dependencies.

**Table 2:** Results of ablation experiment.

Shorter intervals						Longer intervals					
Interval	Model	RMSE	MAPE	RAE	MAE	Interval	Model	RMSE	MAPE	RAE	MAE
15 min	MDFANet	2.70	7.89	9.21	1.22	45 min	MDFANet	5.00	17.22	19.75	2.74
	Without EMB	2.87	7.91	9.45	1.31		Without EMB	5.22	19.95	20.44	2.84
	Without ST	2.70	8.33	9.25	1.28		Without ST	5.10	18.46	19.92	2.76
30 min	MDFANet	4.04	12.55	15.38	2.13	60 min	MDFANet	5.88	22.90	23.89	3.31
	Without EMB	4.25	13.13	15.70	2.18		Without EMB	6.10	25.69	24.62	3.41
	Without ST	4.08	13.85	15.45	2.14		Without ST	6.00	24.63	24.63	3.42

#### Model efficiency

We compared the computational efficiency of MDFANet against the physics-informed baselines (PAG and PIAST) under identical hardware settings. Table 3 reports the end-to-end wall-clock runtime (in seconds) under a fixed epoch budget ($n\_epoch{=}1000$) without early stopping. MDFANet demonstrates a clear advantage in efficiency, requiring only 54.1% of the wall-clock runtime of PAG and 48.8% of PIAST. This efficiency gain is attributed to MDFANet’s aggregated feature representation, which simplifies the model architecture by reducing the complexity typically associated with heavy temporal attention mechanisms or iterative physics-informed constraints.

**Table 3:** Comparison of end-to-end runtime (s) across models.

Model	15 min	30 min	45 min	60 min	Avg (s)
MDFANet	928	939	916	927	928
PIAST	1891	1901	1894	1909	1899
PAG	1716	1714	1722	1710	1716

#### sensitivity evaluation: performance under reduced training data

**Fig. 3:**
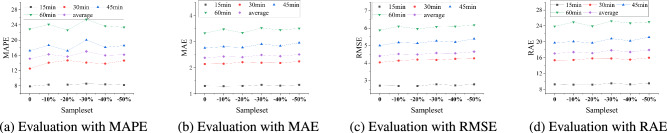
Multi-metric evaluation under progressive data reduction.

To assess the model’s robustness, we evaluated its performance on five subsets of the original data, ranging from 50 to 90% of the full dataset size. As illustrated in Fig. 3, MDFANet shows stable performance across all reduction ratios. Even when training data is reduced by 50%, the increase in MAE is less than 12%, indicating that the performance drop remains limited under this protocol. We note that this experiment reflects sensitivity to training-set size under the above truncation protocol, and we avoid over-generalizing it to other missing-data patterns.

## Conclusion

In this work, we introduced MDFANet to resolve the efficiency-accuracy dilemma in forecasting electric vehicle charging demand. By leveraging a multidimensional feature aggregation module (MDFAM), the model enhances feature representation without the high computational cost of complex temporal attention mechanisms. Quantitative experiments on public datasets validate the superiority of MDFANet, which outperforms state-of-the-art physics-informed baselines (e.g., PIAST) with a MAPE reduction of over 3% and an approximately 50% reduction in end-to-end runtime under the same hardware and a fixed training budget. Additionally, the model shows stable performance under reduced training data, suggesting its potential utility for forecasting-oriented applications as charging networks expand. We note that the current evaluation uses only historical demand and price, and further validation on multimodal datasets that include external factors (e.g., weather, traffic, and grid events) is necessary before making deployment-related claims.

However, this study is subject to certain limitations. The current methodology depends solely on historical charging sequences, neglecting external factors such as weather conditions, traffic dynamics, and grid constraints that can materially affect real-world utility and performance. Moreover, the assumption of static spatial dependencies limits the model’s adaptability to highly dynamic urban topologies where charging station availability fluctuates.

Future work will aim to transcend these boundaries by (1) incorporating multi-modal external data to construct a comprehensive context-aware prediction system, and (2) exploring dynamic graph neural networks to capture evolving spatial correlations, thereby improving the model’s generalization in complex, time-varying urban environments.

## Data Availability

The dataset used during the current study is available in the GitHub repository, https://github.com/IntelligentSystemsLab/ST-EVCDP.

## Footnotes

1 ...
